# Risky sexual behaviours among Ugandan university students: A pilot study exploring the role of adverse childhood experiences, substance use history, and family environment

**DOI:** 10.1371/journal.pone.0277129

**Published:** 2022-11-16

**Authors:** Mark Mohan Kaggwa, Moses Muwanguzi, Sarah Maria Najjuka, Elicana Nduhuura, Jonathan Kajjimu, Mohammed A. Mamun, Innocent Arinaitwe, Scholastic Ashaba, Mark D. Griffiths

**Affiliations:** 1 Department of Psychiatry, Mbarara University of Science & Technology, Mbarara, Uganda; 2 African Centre for Suicide Prevention and Research, Mbarara, Uganda; 3 Department of Psychiatry and Behavioural Neurosciences, McMaster University, Hamilton, Canada; 4 Faculty of Medicine, Mbarara University of Science & Technology, Mbarara, Uganda; 5 Makerere University, College of Health Sciences, Kampala, Uganda; 6 CHINTA Research Bangladesh, Savar, Dhaka, Bangladesh; 7 Department of Public Health and Informatics, Jahangirnagar University, Savar, Dhaka, Bangladesh; 8 Psychology Department, Nottingham Trent University, Nottingham, United Kingdom; University of Nairobi, KENYA

## Abstract

**Background:**

University students are known to have risky sexual behaviours (RSBs). The severity of the RSB is influenced by many factors, including the family environment, exposure to adverse childhood events (ACEs), and the use of addictive substances. However, there is limited information about the influence of ACEs and the family environment of these students in low-and medium-income countries (LMICs). Therefore, a pilot study was conducted among university students from a LMIC, Uganda.

**Methods:**

The present study comprised a cross-sectional online survey among Ugandan students at a public university (N = 316; 75% male; 52.2% aged between 18–22 years). The survey included questions relating to socio-demographic information, family environmental information, the Sexual Risk Survey (SRS), and the Adverse Childhood Experiences-International Questionnaire (ACE-IQ).

**Results:**

Over half (53.8%) reported having had sexual intercourse. Males reported over two times higher mean total SRS score compared to females (*χ*^*2*^ *=* 4.06, *p =* 0.044). Approximately one-sixth of the sample had drunk alcohol or used illicit psychoactive substances in the past six months (16.1%). Among four regression analysis models, sociodemographic variables predicted the highest variance (13%), followed by family environment variables (10%), and both psychoactive substance use history (past six months) and ACEs individually explained approximately 5% variance in total SRS score, with the final model predicting 33% of the variance in RSB.

**Conclusions:**

The present study demonstrated a gender disparity with males involved in more RSB than females, as has been reported in most previous RSB studies. Family environment, sociodemographic factors, substance use, and ACEs all appear to contribute to RSB among university students. These findings will benefit other researchers exploring factors associated with RSB among university students and will help develop interventions to reduce RSB to protect students from unwanted pregnancies, sexually transmitted diseases, and HIV/AIDS.

## 1. Introduction

Among young adults, sexual activity is an important aspect of their lives. Many young people have their first sexual experiences with other individuals when they go to university. In sub-Saharan Africa, the prevalence of individuals having sexual intercourse for the first time at college and university ranges between 22.2% to 52.6% [1, 2]. This is attributed to the university environment having minimal (or no) elder supervision, which offers an opportunity for young people who are transitioning from restrictive adolescence to free and independent adulthood, where they can test their limits of newfound freedom through sexual experimentation [2]. Such sexual exploration involves engagement in risky sexual behaviours (RSBs), including having (i) unprotected vaginal, oral, or anal intercourse, (ii) multiple sexual partners, (iii) inconsistent contraceptive use, and (iv) sexual intercourse under the influence of alcohol [2, 3]. These risky acts are conventionally known to predispose youths to sexually transmitted diseases (especially HIV/AIDS) and unwanted pregnancies [2, 4]. Teenage pregnancies have reduced in most developed high-income regions, but the rates are still high in low-and middle-income countries (LMICs) [5, 6]. Approximately two-thirds of the daily new estimated 6000 new HIV infections globally in 2013 were in sub-Saharan Africa and those were disproportionately found among young women aged 15–24 years [7]. This attribution may be because of the high prevalence of RSB among university students in the region ranging from 26% in Uganda to 63.9% in Botswana [2, 8–11].

Factors that have been associated with RSB among university students include the use of potentially addictive psychoactive substances (i.e., illicit drugs, drinking alcohol), watching pornographic content, poverty, having first sexual intercourse before 18 years, being male, having multiple concurrent sexual partners, peer pressure, living alone, lack of parental control, low family connectedness, poor academic performance, and being a student from urban settings [11–16]. In addition, previous studies in Africa (i.e., Ethiopia) have found significant gender differences where risky sexual practices are higher among males than females [11]. However, females have been reported to have more detrimental consequences, including unwanted pregnancies, sexually transmitted infections (STIs) and complications such as ectopic pregnancies, cervical cancer, chronic pelvic pain, spontaneous abortion, contracting HIV/AIDs, and secondary infertility [17].

Substance use history has consistently been associated with RSB in Uganda [9, 14, 18]. However, the relationship between substance use and RSB is complex and influenced by social, physiological, and individual personality traits [14]. For example, RSB is associated with the impairment in decision-making and reduction in cognitive capacity that causes intoxicated individuals (especially those using alcohol) to focus on the cues that are most salient in the environment (as predicted by alcohol myopia theory), and reduction in the intention to use condoms (as predicted by alcohol expectancy theory) [19–21].

In Uganda, research has shown that most university students are sexually active, start having sex before joining university, have multiple sexual partners at university, rarely use condoms, and engage in sex under the influence of potentially addictive substances, especially alcohol [9, 14]. RSB has been found to be higher among students (i) with lower levels of knowledge about risks involved in premarital and unprotected sex, (ii) studying at mixed-sex secondary schools, and (iii) having one or both parents alive [9]. Consistently, male gender has been associated with higher RSB, especially those having unprotected sex and those who get engaged in sex under the influence of alcohol [14]. In addition, university students affected with depression and anxiety have also been reported to engage in greater RSB [18]. Despite the previous literature, few factors influencing sexual behaviours have been explored in Uganda.

Earlier studies have reported an association between different types of childhood mistreatment (e.g., physical abuse, sexual abuse) and adulthood RSB [22, 23]. In a cross-sectional survey among Polish university students, RSB was associated with physical abuse, emotional abuse and neglect, sexual abuse, and household dysfunction [23]. In the same study, individuals who were sexually abused were six times more likely to have had more than three sexual partners, whereas those who witnessed domestic violence were twice more likely to initiate sexual activity below the age of 16 years [22]. In a retrospective cohort study examining adverse childhood events (ACEs) among adult females in the United States, each category of childhood adversity reported was associated with an increased risk for RSB, and individuals reporting their exposure to at least one ACE reported more RSBs [23]. Experiencing at least four types of ACEs was significantly associated with early sexual initiation and having larger numbers of sexual partners [22]. Among individuals who experienced childhood maltreatment, RSBs appear to attempt to achieve intimate interpersonal relationships and may underestimate the risks they take to achieve intimacy [23]. Based on previous literature, the association between ACEs and RSB may be due to the victim’s desperate attempt to initiate an intimate interpersonal connection. Having grown up in families and/or environments where they could not gain any intimate connections, such individuals may significantly underestimate the risk they are taking to gain intimate connections later on in life through activities like RSBs [23, 24].

Studies in Africa (i.e., Ethiopia and South Africa) investigating the determinants of RSBs among young adults report that single parenting, guardian parenting, and low parental educational level were associated with increased risk for RSB [13, 25]. Other studies in high-income countries (e.g., Sweden), have found that family structure and culture influence sexual behaviour in later life, where dysfunctional families and unstable family environments were associated with first having sexual intercourse at an early age (below 16 years) [26, 27]. In a Rakai community cohort study in Uganda, adolescent girls who headed their households, or lived with stepfathers, grandparents, siblings, or other relatives had a significantly higher prevalence of having sexual intercourse at 16 years or below [28]. Therefore, the family structure appears to be an important correlate of young people’s sexual behaviour. However, a study in Iran reported no relationship between RSB and family structure-related variables such as the number of children in the family, birth order, and family size [29]. The present study further explores the relationship between RSB and family structure-related variables among Ugandan university students. The family structure influence is part of an interlinked system described in Kotchick’s ecological model of determinants of sexual behaviours, namely, the family system (e.g., parenting, parental monitoring, and family socioeconomic status), the self/individual system (i.e., sociodemographic characteristics), and extra-familial system (e.g., peers, partners, school, and neighbourhood) [30].

RSBs have complex determinant mechanisms that range from socio-demographics, childhood adversities, and family environment, which directly influence the risks attached to these sexual behaviours among a young literate population. However, in low-income settings like Uganda, there is still a lack of evidence of the influence of exposure to ACEs and the family environment on the RSBs among university students. Therefore, these factors were explored in the present study. The objectives of the study were to: (i) determine the prevalence of sexual activity among university students and average risky sexual behaviour based on the Sexual Risk Survey (SRS); (ii) determine the different forms of RSBs utilizing the SRS, (iii) examine gender differences in RSBs, and (iv) explore the association between RSBs and socio-demographics, use of psychoactive substances (alcohol and illicit drugs), family environment, and ACEs.

## 2. Methods

### 2.1. Study design, area, and participants

The present pilot study was a cross-sectional online survey conducted among students of Mbarara University of Science and Technology (MUST), a public university in Southwestern Uganda. Data were collected from April 3 to May 23, 2021, using *Google Forms*. The survey link was shared on online platforms like *WhatsApp* groups and personal student emails to students in the university’s six faculties (i.e., Medicine; Computing and Informatics; Business and Management Sciences; Science; Applied Sciences and Technology; and Interdisciplinary Studies), and its two institutions (i.e., Tropical Forest Conservation; and Maternal New-born and Child Health). MUST had over 4,269 undergraduate students enrolled in the academic year 2019/2020, and all were eligible to participate in the study. A total of 316 students participated in the study. The data were collected during the second year of the COVID-19 pandemic when students had just started returning to in-person teaching, and most of the restrictions concerning COVID-19 prevention, such as spatial distancing, had been relaxed. The participants were enrolled using a snowball convenience sampling technique where students who were approached could forward the survey link to other students in the university. To avoid physical contact and to include as many eligible students as possible, snowball convenience sampling was employed to enable efficient recruitment of university students during the COVID-19 pandemic as has been employed in previous studies conducted inside or outside Uganda [31–34].

### 2.2. Data collection

The online survey link was circulated on the different faculty and student social media platforms like end-to-end encrypted *WhatsApp* groups and students’ personal emails. The survey tool was designed to only allow a single response from each student participant. Potential participants received a message requesting them to participate and to share the survey link with their fellow students at MUST. The survey was in English (the language of all teaching in Ugandan universities). Questions were pretested among the students before the commencement of the study to ensure that they were all well understood.

### 2.3. Study measures

The online survey tool included a participant information page, which provided participants with information to understand the intentions of the study, and an informed consent page which all participants completed before responding and participating in the study. As there were no mandatory questions to respond to, participants were free to leave questions unanswered if they were not comfortable and/or sure with the response. However, all participants responded to the questions except one question about the number of sexual partners. In addition, the survey included a sociodemographic questionnaire, family environment questions, the Adverse Childhood Experiences-International Questionnaire (ACE-IQ), and the Sexual Risk Survey (SRS). Given that participants responded to the tool items at their time of convenience, participants were advised to use a calendar of the past six months to accurately remember their past sexual experiences and to minimize memory recall bias (i.e., enhance accurate recall).

#### 2.3.1. Sociodemographic information

Sociodemographic data collected included relevant personal information regarding basic participant characteristics; participant’s age (in years), gender (female, male), marital status (single, separated/divorced, married/cohabiting), and the region of the country of origin (Central, Western, Eastern, and Northern Uganda).

#### 2.3.2. Recent substance use history

A single question (i.e., “*In the past six months have you used alcohol or illicit drugs*?”) with a binary response (yes/no) was used to assess recent substance use history. Those with a ‘yes’ response selected the substances used (i.e., alcohol and/or illicit drugs).

#### 2.3.3. Family environment

Family environment data collected included information on the family type (i.e., nuclear family, extended family, step-parent family, grandparent family, and single parent family); the number of family members; the number of children; primary care provider (i.e., parent, step-parent, uncle/aunt, sibling, guardian, grandparent, NGO, and self-sponsored); birth position in the family; parent’s highest level of education; having a family member with mental illness, or who abuses drugs/substance, or with a criminal record; and whether a parent died before 18 years of age.

#### 2.3.4. Sexual Risk Survey (SRS)

The 23-item SRS [3] was used to assess sexual risk behaviour among college students over a period of six months prior to the study. It comprises five subscales of risky sexual behaviours: sexual risk-taking with uncommitted partners (e.g., *“How many times have you had sex with someone you don’t know well or just met*?*”*), risky sex acts (e.g., *“How many times have you or your partner used alcohol or drugs before or during sex*?*”*), impulsive sexual behaviours (e.g., *“How many times have you had an unexpected and unanticipated sexual experience*?*”*), intent to engage in risky sexual behaviours (e.g., *“How many times have you gone out to bars/parties/ social events with the intent of ‘‘hooking up” and having sex with someone*?*”*) and risky anal sex acts (e.g., *“How many times have you had anal sex without a condom*?*”*) [35], for details, see S1 Table. Raw response frequencies were recorded and converted into ordinal categories which assign weights to the level of sexual risk-taking, ranging from 0 to 4, employing a method used by the scale developers [35]. This approach addresses the skewness of frequency data commonly used in sexual risk assessment studies. The total sexual risk score is calculated as a sum of all raw items’ responses, with total scores ranging from 0 to 92. Higher scores indicate higher sexual risk riskiness. The SRS has shown very good psychometric properties [3], although the Cronbach alpha was 0.69 in the present study. However, the Cronbach alphas for the five subscales were good to excellent: risk-taking with uncommitted partners (α = 0.92), risky sex acts (α = 0.75), impulsive sexual behaviours (α = 0.83), intent to engage in risky sexual behaviours (α = 0.82), and risky anal sex acts (α = 0.82).

#### 2.3.5. Adverse Childhood Experiences-International Questionnaire (ACE-IQ)

The 29-item ACE-IQ [36] was used to assess 13 childhood adversities. Items (e.g., “*During the first 18 years of your life*, *did someone actually have oral*, *anal*, *or vaginal intercourse with you when you did not want them to*?*”*) are responded to on a binary (yes/no) scale. Consequently, total scores range from 0 to 13, where a higher score indicates greater childhood adversity. In previous sub-Saharan African studies, the ACE-IQ has demonstrated good psychometric properties among adolescents and young adults [37–39]. The Cronbach alpha of the ACE-QI in the present study was 0.82.

### 2.4. Ethical considerations

The present study received formal ethical approval from Mbarara University of Science and Technology research ethics committee (MUSTREC#04/01-21). Participants were informed about the sensitive nature of the questions on the SRS and the ACE-IQ due to the potential of some questions to give rise to distressing and negative emotions. Consequently, participants did not have to respond to such questions and were free to end the survey at any point with absolutely no penalty whatsoever. Data confidentiality and anonymity were emphasized. Participation was voluntary, and participants provided informed consent. The survey included a detailed consent form that informed the participants about the study, the risks, and the benefits. All participants were adults who provided their written informed consent to participate in the study; these were automatically granted entry to the study survey. A link to the departmental psychiatry team was provided within the survey, and participants could access the link for help and support if they needed it.

### 2.5. Statistical analysis

Data were imported into STATA Version.15 statistical software, where data were cleaned and analysed. Descriptive statistics are presented in percentages, frequencies, medians, ranges and interquartile ranges. The total score on the SRS and its subscales were analysed as continuous variables. Gender differences in sexual risk-taking and behaviours were assessed by Wilcoxon rank-sum (total scores of SRS and all SRS subscales) and chi-square tests (age at which sexual intercourse first occurred and the number of sexual partners). The Gaussian assumption was used to test for normality of continuous data and was confirmed with Shapiro-Wilks’s test and histograms. Hierarchical Poisson regression was used to determine the predictors of RSBs, and four models were generated. All statistics were calculated at a 95% level of confidence and 5% statistical error.

## 3. Results

### 3.1. Participants’ characteristics

#### 3.1.1. Socio-demographics

A total of 316 students participated in this study. The age ranged from 18 years to 44 years, with over half (52.2%) aged between 18–22 years. The median age was 22 years. Close to three-quarters were males (72.5%), and almost all were single (91.5%) (Table 1).

**Table 1:** Socio-demographics, sexual and family characteristics of participants.

Study variables	Totals (*n = 316*)	
	N	
**Socio-demographics**		
**Age (in years)**		
18–22	165	52.2%
23–44	151	47.7%
**Gender**		
Female	87	27.5%
Male	229	72.5%
**Marital status**		
Single	289	91.5%
Married/cohabiting	24	7.6%
Separated/divorced	3	0.95%
**Region of origin**		
Central	86	27.2%
Eastern	45	14.2%
Northern	19	6.0%
Western	166	52.5%
**Year of Study**		
First	63	19.9%
Second	112	35.4%
Third	55	17.4%
Fourth	55	17.4%
Fifth	31	9.8%
**Substance use**		
**History of substance use (alcohol and/or illicit drugs) in the past six months**		
No	265	83.9%
Yes	51	16.1%
**History of alcohol use in the past six months**		
No	272	86.1%
Yes	44	13.9%
**History of illicit drug use in the past six months**		
No	290	91.8%
Yes	26	8.2%
**Family environment**		
**Type of family**		
Extended family	97	30.7%
Grandparent family	6	1.9%
Nuclear family	163	51.6%
Single parent family	38	12.0%
Step-family	12	3.8%
**Primary care provider**		
Parent	249	78.8%
Step-parent	2	0.6%
Uncle/aunt	15	4.8%
Sibling	12	3.8%
Guardian	18	5.7%
Grandparent	10	3.2%
NGO	7	2.2%
Self-sponsored	3	1.0%
**Number of family members**		
0–6	101	32.0%
7–8	89	28.2%
9–10	65	20.6%
11–25	61	19.3%
**Number of children in the family**		
0–4	83	26.3%
5–6	105	33.2%
7–8	68	21.5%
9–18	60	19.0%
**Birth position**		
1^st^	77	24.4%
2^nd^	57	18.0%
3^rd^	69	21.8%
4^th^ or lower	113	35.8%
**Family member with mental illness**		
Yes	48	15.2%
No	268	84.8%
**Family member with substance use disorder**		
Yes	101	32.0%
No	215	68.0%
**Family member with a criminal record**		
Yes	53	16.8%
No	263	83.2%
**Parent died before 18 years**		
Yes	73	23.1%
No	243	76.9%
**Mother’s education level**		
None	58	18.4%
Primary	77	24.4%
Secondary	91	28.8%
Tertiary	90	28.5%
**Father’s education level**		
None	47	14.9%
Primary	45	14.2%
Secondary	79	25.0%
Tertiary	145	45.9%
**Adverse childhood experiences**		
**Number of ACEs**		
0	0	0%
1	2	0.6%
2	14	4.4%
3	33	10.4%
≥ 4	267	84.5%
**Physical abuse**		
No	0	0%
Yes	316	100%
**Emotional abuse**		
No	102	32.3%
Yes	214	67.7%
**Contact sexual abuse**		
No	222	70.3%
Yes	94	29.7%
**Alcohol and/or drug abuser in the household**		
No	220	69.6%
Yes	96	30.4%
**Incarcerated household member**		
No	255	80.7%
Yes	61	19.3%
**Someone chronically depressed, mentally ill, institutionalized or suicidal in family**		
No	258	81.7%
Yes	58	18.3%
**Household member treated violently**		
No	81	25.6%
Yes	235	74.4%
**One or no parents, parental separation or divorce**		
No	161	51.0%
Yes	155	49.1%
**Emotional neglect**		
No	101	32.0%
Yes	215	68.0%
**Physical neglect**		
No	226	71.5%
Yes	90	28.5%
**Bullying**		
No	202	63.9%
Yes	114	36.1%
**Community violence**		
No	44	13.9%
Yes	272	86.1%
**Collective violence**		
No	179	56.6%
Yes	137	43.4%

*Abbreviations***:** ACEs = Adverse Childhood Experiences, NGO = Non-Governmental Organization

#### 3.1.2. Substance use

Approximately one-sixth of the sample (16.1%; n = 70) reported a recent history of psychoactive substance use (i.e., alcohol and/or illicit drugs in the past six months). The majority had a history of drinking alcohol (62.9%, 44/70) (Table 1).

#### 3.1.3. Family characteristics

Regarding the family environment, approximately half of the participants resided in nuclear families (51.6%), with approximately four-fifths having parents as their primary care providers (78.8%). It was also found that just over one-sixth reported living with a family member with mental illness (15.2%), and about one-third lived with a family member with substance use disorder (32%) (Table 1).

#### 3.1.4. Adverse childhood experiences

The mean score on the ACE-IQ was 6.5 (SD±2.6). All participants reported exposure to at least one ACE, with the majority of participants (84.5%) reporting experiencing four or more childhood adversities. Moreover, all participants reported having been physically abused (100%) and just below one-third reported having been sexually abused (29.7%). Approximately one-fifth reported having had a household member sent to prison (19.3%) and/or a family member who was mentally ill (18.3%) (Table 1).

### 3.2. Risky sexual behaviours

Over half of the student sample reported that they had engaged in sexual intercourse (53.8%), with one-fifth reporting having had more than one sexual partner (19.6%) (Table 2). The mean score of the total SRS was 13.4 (±SD = 14.6). The riskiest types of sexual behaviours reported by students were having sex with uncommitted partners (mean = 5.33; SD = 7.14), and the least risky types of sexual behaviour reported were anal sexual behaviours (mean = 0.34; SD = 1.20). The mean average scores for the other risky sexual behaviours were 1.81 (SD = 2.55) for risky sex acts, 4.87 (SD = 4.79) for impulsive sexual behaviours, and 1.09 (SD = 1.88) for intent to engage in risky sexual behaviours.

**Table 2:** Gender differences in sexual risk-taking and behaviours.

Sexual risky behaviour	Total (median, IQR)	Gender		*χ* ^*2*^	*p-*value
		Female (median, IQR)	Male (median, IQR)		
Sexual risk-taking with uncommitted partners (F_1_)	2 (8)	1 (6)	3 (10)	2.79	0.095
Risky sex acts (F_2_)	1 (3)	0 (2)	1 (3)	3.05	0.081
Impulsive sexual behaviours (F_3_)	4 (4)	4 (6)	3 (7)	0.09	0.757
Intent to engage in risky sexual behaviours (F_4_)	0 (2)	0 (0)	0 (2)	9.72	**0.002**
Risky anal sex (F_5_)	0 (0)	0 (0)	0 (0)	0.33	0.567
Total sexual risk score	8.5 (19)	5 (18)	11 (19)	4.06	**0.044**
**Age of first sexual intercourse: n (%)**					
Not yet	146 (46.2%)	47 (54.0%)	99 (43.23%)	12.22	**0.016**
Younger than 13 years	17 (5.4%)	6 (6.9%)	11 (4.80%)		
13–15 years	14 (4.4%)	0	14 (6.1%)		
16–17 years	25 (7.9%)	2 (2.3%)	23 (10.0%)		
18 years or older	114 (36.1%)	32 (36.8%)	82 (35.8%)		
* **Number of sexual partners (n = 276) n (%)**					
None	124 (39.2%)	43 (55.1%)	81 (40.9%)	5.27	0.072
One	90 (28.5%)	23 (29.5%)	67 (33.8%)		
More than one	62 (19.6%)	12 (15.4%)	50 (25.3%)		

* Nine individuals did not respond to the question; IQR = Interquartile range

#### 3.2.1. Gender differences in risky sexual behaviour

Generally, males had higher median SRS scores across all subscales and had wider interquartile ranges than females. The median total SRS score for males was over two times higher than that for females (*χ*^*2*^ *=* 4.06, *p =* 0.044). Among all subscales, males had a higher score on the ‘Intent to engage in risky sexual behaviours’ subscale than females (*χ*^*2*^ *=* 9.72, *p =* 0.002). There was a statistically significant difference between the age at which individuals first had sexual intercourse and gender (*χ*^*2*^ *=* 12.22, *p* = 0.016), with more females first having intercourse at the extreme of the age groups (below 13 years, and 18 years and above) than males (Table 2).

### 3.3. Predictive models of risky sexual behaviour severity

Table 3 presents a multiple hierarchical Poisson regression analysis with a total of four models for predicting RSBs among the sample. Model 1 includes all the sociodemographic variables, which predicted approximately 13% of the variance in RSB. All the sociodemographic variables exhibited a significant relationship with the SRS score. The explained variance in RSB increased to approximately 18% in the second model after adding the substance use variable (illicit drug use and drinking alcohol in the past six months). The addition of family environment factors into the third model increased the variance in RSB to 28%. In the final model, adverse childhood experiences were added, and the variance in RSB increased by approximately 5%. All the models were significant, and the final model accounted for 33.33% variance in predicting RSB.

**Table 3:** Predictive models for risky sexual behaviour (n = 316).

Variable		Model 1	Model 2	Model 3	Model 4
		*χ*^*2*^ = 784.04	*χ*^*2*^ = 1084.62	*χ*^*2*^ *=* 1665.58	*X*^*2*^ *=* 1956.47
		Pseudo-R^2^ = 0.1336	Pseudo-R^2^ = 0.1848	Pseudo-R^2^ = 0.2838	Pseudo-R^2^ = 0.3333
		*p*<0.001	*p*<0.001	*p*<0.001	*p<*0.001
		b (95% C I)	b (95% C I)	b (95% C I)	b (95% C I)
**Constant**		2.79 (2.49–3.09) **	2.08 (1.77–2.38) **	.66 (.16–1.16) *	1.63 (1.08–2.17) **
**Socio-demographics**	Age	.02 (-.01–.03) **	.03 (.02–.04) **	.03 (.02–.05) **	-.01 (-.01–.01)
	Male gender ^x^	0.39 (.31–.47) **	.30 (.23–.38) **	.25 (.16 –.34) **	.33 (.24–.43) **
	Marital Status ^a^				
	Separated/Divorced	0.77 (0.55–0.99) **	0.80 (0.58–1.02) **	1.13 (.87–1.39) **	0.49 (.20–.78) *
	Single	-0.61 (-0.72–- 0.49) **	-0.32 (-0.43–-0.20) **	-.45 (-.60–-.31) **	-.60 (.75–-.44) **
	Region ^b^				
	Eastern	-0.20 (-0.30–-0.10) **	-.22 (-.32–-.11) **	-.44 (-.57–-.31) **	-.49 (.63–-.35) **
	Northern	-1.33 (-1.54–- 1.12) **	-1.33 (-1.54–-1.11) **	-1.47 (-1.69–-1.24) **	-1.38 (-1.62–-1.15) **
	Western	-0.23 (-0.30–-0.16) **	-.26 (-.33–-.19) **	-.30 (-.39–-.22) **	-0.27 (-0.36–-.17) **
	Year of study ^c^				
	Second	-0.19 (-0.28–-0.11) **	-.21 (.29–-.12) **	-.21 (-.31–-.11) **	.03 (-.08–.14)
	Third	0.17 (0.07–0.26) **	0.16 (0.64–0.25) *	.40 (.29–.51) **	.50 (.39–.62) **
	Fourth	-0.28 (-0.38–-0.17) **	-0.46 (-.57–-.36) **	-.37 (-.49–-.25) **	-.08 (.21–.05)
	Fifth	-0.75 (-0.89–-0.61) **	-.67 (-.81–.52) **	-.93 (-1.11–-.76) **	-.61 (-.79–-.43) **
**History of substance use (past six months)**	History of substance use ^y^		.67 (.60–.74) **	.52 (-.43–-.61) **	.24 (.14–.35) **
**Family environment**	Type of family ^d^				
	Grandparent family			-.45 (-.81–-.08) *	-.21 (-.59–.17)
	Nuclear family			.15 (.07–.24) **	.19 (.10–.28) **
	Single parent family			-.14 (-.27–-.01) *	.01 (-.14–.14)
	Stepfamily			-1.04 (-1.29–-.79) **	-1.35 (-1.62–-1.07) **
	Primary care provider ^e^				
	Step-parent			1.97 (1.59–2.36) **	1.85 (1.45–2.25) **
	Uncle/aunt			-.03 (-.21–.15)	.12 (-.08–.32)
	Sibling			.52 (.35–.70) **	.33 (.14–.53) *
	Guardian			-.42 (-.62–-.22) **	-.18 (-.39–.03)
	Grandparent			-.38 (-.67–-.09) *	-.61 (-.93–-.30) **
	NGO			1.23 (1.04–1.42) **	1.52 (1.30–1.74) **
	Self-sponsored			.75 (.53–.96) **	.61 (.38–.85) **
	Number of family members			.01 (.01-.03) *	.01 (.00–.02) ^z^
	Number of children in the family			.01 (.01-.02)	.01 (-.01–.03)
	Birth position ^f^				
	2^nd^			-.29 (-.40–-.18) **	-.32 (-.43–-.21) **
	3^rd^			-.39 (-.49–-.28) **	-.32 (-.43–-.20) **
	4^th^ or lower			-.18 (-.28–-.08) **	-.16 (-.27–-.05) *
	Family with mental illness ^y^			.11 (.01–.23) *	.02 (-.09–.14) *
	Family with substance abuse ^y^			.18 (.10–.26) **	.08 (-.02–.18)
	Family with criminal record ^y^			.22 (.13–.32) **	.13 (.01–.24) *
	Lost parent before 18 years ^y^			.14 (.04–.23) *	-.07 (-.19–.05)
	Mother’s education level ^g^				
	Primary			-.16 (-.29–-.03) *	-.18 (-.31–-.04) *
	Secondary			.26 (.14–.38) **	.25 (.11–.39) **
	Tertiary			.15 (.02–.28) *	-.05 (-.20–.10)
	Father’s education level ^g^				
	Primary			.01 (-.14–.16)	.19 (.02–.36) *
	Secondary			-.12 (-.26–.02)	-.02 (-.17–.13)
	Tertiary			-.13 (-.26–-01) *	.08 (-.07–.23)
**Adverse childhood events**	Physical abuse ^y^				Omitted
	Emotional abuse ^y^				.09 (.01–.19) *
	Contact sexual abuse ^y^				.21 (.11–.32) **
	Alcohol and/or drug abuser in the household ^y^				.26 (.17–.36) **
	Incarcerated household member ^y^				-.22 (-.34–-.11) **
	Someone chronically depressed, mentally ill, institutionalized or suicidal ^y^				.20 (.09–.31) **
	Household member treated violently ^y^				-.09 (-.19–.01)
	One or no parents, parental separation or divorce ^y^				-.16 (-.25–-.06) *
	Emotional neglect ^y^				.19 (.10–.27) **
	Physical neglect ^y^				.20 (.11–.29) **
	Bullying ^y^				.26 (.17–.34) **
	Community violence ^y^				-.13 (-.24–-.02) *
	Collective violence ^y^				.20 (.11–.30) **

* p<0.05;

** p<0.01,

^z^ p = 0.05

Abbreviations were defined as:

b = beta coefficient; CI = Confidence Interval

Reference:

^a^ = Married/Cohabiting,

^b^ = Central,

^c^ = year one

^d^ = Extended family

^e^ = Parent,

^f^ = first born

^g^ = None,

^x^ = female,

^y^ = No

Among the sociodemographic variables, the following factors increased RSB score: male gender, being separated/divorced, and being a third-year university student. While being single, coming from a region other than the central region, and being a fifth-year university student increased the RSB score. History of the use of psychoactive substances increased the RSB score in the final model. For family environment-related factors, being raised in a nuclear family, the type of primary care provider (i.e., being a step-parent, sibling, NGO, and self-sponsored), family history of mental illness, family member having a criminal record, father having a primary level of highest education, and mother having a secondary level of highest education increased the RSB score among university students. However, being raised in a grandparent and step-parent type of family, grandparent being the primary care provider, not being the first-born child in the family, and mother has a primary level of education resulted in a significantly reduced RSB score among university students. Almost all ACEs were statistically significantly associated with RSB scores except for having a family member treated violently. The majority of the ACEs increased the RSB score (i.e., emotional abuse, contact sexual abuse, alcohol and/or drug abuse in the household, someone being chronically depressed, mentally ill, institutionalized, or suicidal in the family, emotional neglect, physical neglect, bullying, and collective violence) except incarceration of a household member and community violence (Table 3). There was no collinearity among the variables included in the final model (mean VIF of 1.64 and all the individual VIFs were below 3).

## 4. Discussion

The present study investigated the prevalence of sexual activity among university students, and risky sexual behaviours were assessed as a continuous variable using the Sexual Risk Survey (SRS). The study is the first to be conducted in Uganda that used a population-specific standardized assessment tool to study risky sexual behaviours (RSBs) among university students. Additionally, the study analysed the (i) different forms of RSBs utilizing the SRS, (ii) gender differences in RSBs, and (iii) association between RSBs and socio-demographics, use of psychoactive substances (alcohol and illicit drugs), family environment, and adverse childhood experiences. The few previous studies conducted among Ugandan university students have only assessed RSBs, such as the age at which sexual intercourse first took place (≤16 years), having multiple sexual partners, and inconsistent condom use [9, 14], which is very narrow compared to the present study.

The mean score of the total SRS of 13.4 was lower than that found among the US studies using the same tool: 16.19 reported by Wang et al. (2018) [40]; 14.7 reported by Turchik et al. (2015) [35], and 16.3 reported by Hahn, Simons, and Simons, (2016) [41]. This lower score may be because Uganda is an HIV epidemic region, and its university students, especially females, may take fewer risks due to fear of contracting HIV [42]. In addition, the 23-item SRS has not been validated for use among Ugandan university students. Some questions may not have been completely understood by all students resulting in lower scores. For example, some words (e.g., ‘cunnilingus’, ‘dental dam’) may not be familiar to those completing the survey and require individuals to use a guide or glossary to understand them [3]. The present study found that over half of university students reported having sexual intercourse at the time of the study (53.8%), which was higher than that reported among University students in Ethiopia, ranging between 26.9% and 38.9% [13, 43, 44]. In a secondary analysis of Global School-based Student Health Surveys of five sub-Saharan counties (i.e., Benin, Mozambique, Namibia, Seychelles, and Tanzania), the overall prevalence of ever having had sexual intercourse was 43.5% [45], which is slightly lower than that in the present study. Moreover, in another study, 88.5% of young adults attending higher learning institutions in Mbeya (Tanzania) were sexually active [46], which is higher than that reported in most sub-Saharan countries. The variations in the prevalence rates may be due to differences in participant characteristics and, more importantly, the cultural variations and social norms regarding sexual activity in African countries [47]. In addition, there may be differences in college-based sexual risk education and information provision to students, which could also be a reason for such variations [47].

Most studies conducted among university and college students have found that males are more engaged in risky sexual behaviours than females [40, 48, 49]. In the present study, males had higher RSBs than females, and being male statistically significantly increased RSB score. However, such findings may be due to sexual double standards as well as social desirability biases. For example, a study among private college students in Mekelle City (North Ethiopia) reported that males were four times more likely to have multiple sexual partners than females [43]. Additionally, in a systematic review of the epidemiology of sexual behaviours among Ethiopian college and university students, males were 2.35 times more likely to engage in RSBs compared to females [11]. This may be due to cultural influences where it is socially acceptable for men to take charge in sexual relationships, and increase the likelihood of having multiple sexual partners. Similarly, it may be due to premarital sexual permissiveness attitudes, which are more common among males than females [50].

The present study also found that anal sex was not frequently reported among the student sample surveyed. Anal intercourse is not socially acceptable in Uganda and other parts of Africa, perhaps explaining the low rates reported in the study [51–53]. In qualitative studies exploring perceptions of’ heterosexual penile-anal intercourse among women in three sub-Saharan African countries (i.e., Zimbabwe, Uganda, and South Africa), participants described this socially stigmatized act as disgusting, embarrassing, sinful, and shameful [51, 52]. They also added that this type of sexual activity is against cultural and religious morals and that such acts may be practiced only by sex workers, drug addicts, and porn stars. Across the three countries, participants asserted that anal sex was only practiced by perverse, ‘messed-up’, ‘insane’, or mentally ill people [51]. Therefore, negativism and stigmatization of anal sexual practices do not appear to be practiced very often, and/or individuals do not report it even if they engage in such practices.

The present study generated four statistically significant models to predict risky sexual behaviours in the past six months, based on students’ sociodemographic factors, substance use history in the past six months (alcohol and/or illicit drugs), family environment, and adverse childhood experiences. All models were statistically significant and indicated the importance of these factors in explaining RSBs among university students, they only explained 33% of the variance. This suggests that other factors play a bigger role in RSBs among university students. However, the findings in the present study are similar to those reported in many other studies in African countries such as Botswana, Ethiopia, and Nigeria [11, 54, 55]. However, due to the exploratory nature of the present study, the models generated arguably have limited comparison with other prior African studies.

Substance use history was associated with an increase in RSBs in the present study, a finding that has consistently been reported in other Ugandan studies [9, 14, 18]. Use of potentially addictive psychoactive substances has been associated with impulsivity, impaired decision-making, reduction in cognitive capacity, and causes people to focus on the cues that are most salient in the environment (as predicted by alcohol myopia theory), and reduction in the intention to use condoms especially following alcohol drinking (as predicted by alcohol expectancy theory) [19–21]. The relationship between psychoactive substance use and RSB is complex and influenced by social, physiological, and individual personality traits [14]. Therefore, the use of psychoactive substances should be managed holistically to avoid its complications such as RSBs.

Due to rural-urban migration and increased cost of living, Uganda’s families are becoming nuclear [56]. However, these were associated with an increase in RSB in the present study, a finding similar to previous research, which reported less frequent condom use and early onset of sexual life among children brought up in nuclear families [57, 58]. This finding was attributed to students from nuclear families not respecting traditions such as sharing family dinners–where most important discussions are started in families [57]. The aspect of sharing and respecting traditions is still present in the grandparent family type where traditions are still followed, which may explain its association with reducing RSB in the present study. Surprisingly, growing up in step-families was also associated with reducing RSB, a finding contradictory to previous studies [59]. This may be due to a fear of these students getting involved in RSB due to fear of getting pregnant and producing children, who may, unfortunately, end up as stepchildren due to fear of relationship stability–since some grow up in a family where a relationship failed (their parents could not stay with their children) and they ended up as stepchildren. Students whose parents or grandparents are not their university tuition fees sponsors go through many hardships to obtain tuition fees. Since it is culturally believed that only grandparents and parents usually provide unconditional support without the expectation of being paid back and may easily offer money to students for their daily expenses while at university. Many students not sponsored by their grandparents or parents may get involved in RSBs, either to acquire extra money or cope with their stress [60]. Similar to other studies [13, 27], the highest level of education of the parents was associated with higher RSB scores among university students in the present study (i.e., the father has a primary level and the mother has a secondary level of education). However, students having mothers with a primary level of education were associated with lower RSB scores. Mothers who have a minimal level of education may be very scared about their children at university being exposed to risky behaviours and may provide better advice compared to those with a secondary level of education who missed going to university, who may want their children to enjoy university and may advise them to have fun (which may result on higher RSB scores among their children). Fathers with a primary level of education may feel intimidated by their children who are at university and may fail to advise them against getting involved in RSBs.

Not being the first-born child in the family was significantly associated with reduced RSB scores among university students. This could be due to them getting advice from elder siblings or learning from the complications faced by elder siblings due to involvement in RSB. More family-related factors, such as the family history of mental illness and a family member having a criminal record, were significantly associated with higher RSB scores. The pair (i.e., a family member with a criminal record and family history of mental illness) has been associated with ACEs, and many classify them as a form of ACE, which has consistently been associated with RSB in adulthood [23, 61]. Researchers have suggested that ACEs significant association with RSB is due to victims desperately attempting to initiate and maintain an intimate interpersonal connection [23]. Since they grow up in families and/or an environment that was unable to protect them and/or have intimate connections, it may significantly make them underestimate the risk they are taking to achieve intimate connections or protection and may end up involved in RSB [23, 24]. Because of these possible associations between ACEs and RSBs, the majority of the ACEs were significantly associated with higher RSB scores, including emotional abuse, contact sexual abuse, alcohol and/or drug abuse in the household, someone being chronically depressed, mentally ill, institutionalized, or suicidal in the family, emotional neglect, physical neglect, bullying, and collective violence. However, incarceration of a household member and community violence were significantly associated with a lower RSB score. In the case of incarceration of a household member, if the member was abusive, then the child (now the university student) could have felt protected–an aspect associated with lower involvement in RSB [23, 24]. For the case of community violence, more research is needed to understand this paradoxical result. Interestingly, all participants in the present study had experienced physical abuse before the age of 18 years. This may be attributed to the parental style commonly used in Uganda that involves physically punishing children to discipline them (i.e., corporal punishment) [62, 63]. A comparative study with students who have not experienced a similar parenting style involving corporal punishment would enable a better understanding of the relationship between childhood physical abuse and RSB.

## 5. Limitations and future research

The present study had some limitations that should be considered when interpreting the findings reported here (as well as taking these on board in developing future research). Due to its cross-sectional nature, the study is not able to draw causal inferences. Future research should therefore use longitudinal designs to delineate the causality between the variables studied here. In addition, the study did not capture all sexual-related factors such as sex-related alcohol consumption and condom use. Therefore, future studies should incorporate these and other factors that help determine the factors that contribute to RSB. The SRS arguably provided a comprehensive assessment of participants’ sexual activity, but future research could expand on the five scale domains and examine other types of RSB not included (e.g., risky paraphilic behaviour).

Another limitation was that the present study had a large minority of participants who had never engaged in sex at all (approximately 46%) which means the factors underlying RSBs were from a smaller subsample of all participants. However, Schuster et al. (1996) reported that among virgins, there is still a likelihood of involvement in genitally-based sexual practices, including fellatio, and cunnilingus, which increase the likelihood of transmission of STIs, including HIV/AIDS [64]. Further research is needed among virgins to establish the types of RSB they may engage in even if they are not having sexual intercourse.

The present study only comprised students from one Ugandan university, which may not be representative of other Ugandan university students. Therefore, future research using more representative samples of Ugandan university students is needed to replicate and confirm the findings in the present study. It should also be noted that since the study used snowball sampling to recruit students, it cannot be guaranteed that all responses were university students from the same university. Therefore, future research on the student population needs to implement methods that can confirm the student status of participants.

Because the present study examined a very sensitive topic (i.e., risky sexual behaviour), the responses are likely to have had social desirability biases, and the retrospective nature of the study may have included recall biases (although participants were advised to only report sexual activity in the past six months). Therefore, future research is needed to qualitatively assess the lived experiences and factors that facilitate or inhibit risky sexual behaviours among university students to develop specific interventions to reduce unplanned pregnancies, STIs, and the spread of HIV/AIDS.

It should also be noted that the Cronbach’s alpha on the SRS was relatively low and the instrument has not been previously validated among Ugandan university students or similar cultural populations (e.g., sub-Sahara African counties), and some of the questions may not have been culturally appropriate. Consequently, future studies should validate the tool for university students in sub-Saharan Africa. Moreover, in the present study, substance use severity and specific types of illicit psychoactive substance use were not assessed in detail and only one item in the study related to substance use. Given that substance use appeared to be an important correlate with sexual behaviour, future studies should look at this variable in more depth to include the range of substances used as well as the severity of substance use disorders.

When considering all the study variables, the sample size was modest. This could have affected the robustness of the final model. Therefore, future studies should involve larger samples to investigate more rigorously the relationships between the studied variables and RSBs. Finally, the results concerning RSB reported in the present study may not be a true reflection of the student’s behaviours since the previous 12 months, they were not at university due to the COVID-19 pandemic and its associated restrictions (e.g., lockdowns). Also, the high level of depression and its associated symptoms, such as lack of motivation, anhedonia, hypersomnia, among students during the first months of the pandemic [33] may have resulted in fewer students being involved in RSBs. Therefore, replication of this study in a non-pandemic period is needed to ascertain whether any of the present study’s findings were affected by pandemic-related factors.

## 6. Conclusion

As with many previous studies, the present study demonstrated that most Ugandan undergraduate university students had engaged in sexual activity and that riskier sexual practices were more likely among males than females. Sociodemographic factors, history of substance use, family environment, and adverse childhood experiences appear to have a role in risky sexual behaviours among Ugandan university students. Although the present study’s findings are preliminary, they can be used as a basis for other researchers to explore these and other factors associated with risky sexual behaviour among university students. Identifying the factors in RSBs will ultimately help develop interventions to reduce such behaviour so that students can be protected from unwanted pregnancies, STIs, and HIV/AIDS. Based on such findings, awareness-building programs among students in LMICs could be developed focusing on safe sexual practices and where to seek help if someone is already engaging in RSBs. Such programs should be implemented based on evidence-based health promotion theories such as the Theory of Planned Behaviour and the Health Belief Model [65–67].

## Supporting information

S1 Table Sexual Risk Survey (SRS) items and converting raw scores to ordinal categories. (DOCX)

## References

[1] Gelibo T, Belachew T, Tilahun T. Predictors of sexual abstinence among Wolaita Sodo University Students, South Ethiopia. Reproductive Health. 2013;10(1):18. doi: 10.1186/1742-4755-10-18 23547969 PMC3636112

[2] Shiferaw Y, Alemu A, Assefa A, Tesfaye B, Gibermedhin E, Amare M. Perception of risk of HIV and sexual risk behaviors among University students: Implication for planning interventions. BMC Research Notes. 2014;7(1):162. doi: 10.1186/1756-0500-7-162 24642193 PMC3974211

[3] Turchik JA, Garske JP. Measurement of sexual risk taking among college students. Archives of Sexual Behavior. 2009;38(6):936–48. doi: 10.1007/s10508-008-9388-z 18563548

[4] Rutherford GW, Anglemyer A, Bagenda D, Muyonga M, Lindan CP, Barker JL, et al. University students and the risk of HIV and other sexually transmitted infections in Uganda: the Crane survey. International Journal of Adolescent Medicine and Health. 2014;26(2):209–15. doi: 10.1515/ijamh-2013-0515 24762640

[5] World Health Organisation. Adolescent pregnancy 2018. https://www.who.int/en/news-room/fact-sheets/detail/adolescent-pregnancy.

[6] Finer LB, Zolna MR. Declines in unintended pregnancy in the United States, 2008–2011. New England Journal of Medicine. 2016;374(9):843–52. doi: 10.1056/NEJMsa1506575 26962904 PMC4861155

[7] Kharsany ABM, Karim QA. HIV infection and AIDS in sub-Saharan Africa: current status, challenges and opportunities. The Open AIDS Journal. 2016;10:34–48. doi: 10.2174/1874613601610010034 27347270 PMC4893541

[8] Omoteso BA. A study of the sexual behaviour of university undergraduate students in southwestern Nigeria. Journal of Social Sciences. 2006;12(2):129–33. doi: 10.1080/09718923.2006.11978380

[9] Musiime K, Mugisha J. Factors associated with sexual behaviour among students of Uganda Martyrs University. International Journal of Public Health Research. 2015;3:85–93.

[10] Hoque ME, Ntsipe T, Mokgatle-Nthabu M. Sexual practices among university students in Botswana. Gender and Behaviour. 2012;10(2):4645–56. 10.1080/17290376.2013.869649 PMC3914499 24405283

[11] Amare T, Yeneabat T, Amare Y. A systematic review and meta-analysis of epidemiology of risky sexual behaviors in college and university students in Ethiopia, 2018. Journal of Environmental and Public Health. 2019:4852130. doi: 10.1155/2019/4852130 31015844 PMC6446110

[12] Noubiap JJN, Nansseu JRN, Ndoula ST, Wang B, Jingi AM, Bigna JJR, et al. Prevalence and correlates of HIV-risky sexual behaviors among students attending the medical and social welfare center of the University of Maroua, Cameroon. BMC Research Notes. 2015;8(1):635. doi: 10.1186/s13104-015-1638-2 26526854 PMC4630846

[13] Fentahun N, Mamo A. Risky sexual behaviors and associated factors among male and female students in Jimma Zone preparatory schools, South West Ethiopia: comparative study. Ethiopian Journal of Health Sciences. 2014;24(1):59–68. doi: 10.4314/ejhs.v24i1.8 24591800 PMC3929929

[14] Choudhry V, Agardh A, Stafström M, Östergren P-O. Patterns of alcohol consumption and risky sexual behavior: A cross-sectional study among Ugandan university students. BMC Public Health. 2014;14(1):128. doi: 10.1186/1471-2458-14-128 24502331 PMC3933239

[15] Mehra D, Kyagaba E, Ostergren PO, Agardh A. Association between self-reported academic performance and risky sexual behavior among Ugandan university students—a cross sectional study. Global Journal of Health Science. 2014;6(4):183–95. doi: 10.5539/gjhs.v6n4p183 24999121 PMC4825383

[16] Mehra D, Agardh A, Stafström M, Östergren PO. Is drinking alcohol associated with sexual coercion among Ugandan university students?: A cross-sectional study. Reproductive Health. 2014;11(1):7. doi: 10.1186/1742-4755-11-7 24438109 PMC3901843

[17] World Health Organisation. Sexually transmitted infections (STIs) 2021 [cited 2021 1 december]. https://www.who.int/news-room/fact-sheets/detail/sexually-transmitted-infections-(stis).

[18] Kintu TM, Kaggwa MM, Namagembe R, Muganzi DJ, Kihumuro BR, Luyinda GS, et al. Alcohol use disorder among healthcare professional students: A structural equation model describing its effect on depression, anxiety, and risky sexual behavior. 2022. Research Square (preprint). 10.1186/s12888-023-04989-1 PMC10339521 37438721

[19] Weinhardt LS, Carey MP. Does alcohol lead to sexual risk behavior? Findings from event-level research. Annual Review of Sex Research. 2000;11:125–57. 11351830 PMC2426779

[20] Brown JL, Gause NK, Northern N. The association between alcohol and sexual risk behaviors among college students: A review. Current Addiction Reports. 2016;3(4):349–55. doi: 10.1007/s40429-016-0125-8 27896039 PMC5123847

[21] Wagenaar C, Florence M, Adams S, Savahl S. Factors influencing the relationship between alcohol consumption and risky sexual behaviour among young people: A systematic review. Cogent Psychology. 2018;5(1):1483049. doi: 10.1080/23311908.2018.1483049

[22] Makaruk K, Włodarczyk J, Sethi D, Michalski P, Szredzińska R, Karwowska P. Survey of adverse childhood experiences and associated health-harming behaviours among Polish students. Copenhagen: World Health Organization. Regional Office for Europe, 2018 2018. Report No.: Contract No.: WHO/EURO:2018-2984-42742-59621.

[23] Hillis SD, Anda RF, Felitti VJ, Marchbanks PA. Adverse childhood experiences and sexual risk behaviors in women: A retrospective cohort study. Family Planning Perspectives. 2001;33(5):206–11. 11589541

[24] Strasburger VC, Donnerstein E. Children, adolescents, and the media: Issues and solutions. Pediatrics. 1999;103(1):129–39. doi: 10.1542/peds.103.1.129 9917450

[25] Mmari K, Kalamar AM, Brahmbhatt H, Venables E. The influence of the family on adolescent sexual experience: A comparison between Baltimore and Johannesburg. PloS One. 2016;11(11):e0166032. doi: 10.1371/journal.pone.0166032 27820853 PMC5098750

[26] Kastbom Å A, Sydsjö G, Bladh M, Priebe G, Svedin CG. Differences in sexual behavior, health, and history of child abuse among school students who had and had not engaged in sexual activity by the age of 18 years: a cross-sectional study. Adolescent Health, Medicine and Therapeutics. 2016;7:1–11. doi: 10.2147/AHMT.S95493 26811695 PMC4712967

[27] Asamoah BO, Agardh A. Individual- and family-level determinants of risky sexual behavior among Swedish- and foreign-born young adults 18–30 years of age, residing in Skåne, Sweden. Archives of Sexual Behavior. 2018;47(2):517–28. doi: 10.1007/s10508-017-0978-5 28560591 PMC5775364

[28] Pilgrim NA, Ahmed S, Gray RH, Sekasanvu J, Lutalo T, Nalugoda F, et al. Family structure effects on early sexual debut among adolescent girls in Rakai, Uganda. Vulnerable Children and Youth Studies. 2014;9(3):193–205. doi: 10.1080/17450128.2013.842027 25317199 PMC4194054

[29] Rezazadeh M, Ahmadi K, Nafarieh M, Akhavi Z, Zanganeh MA, Maoudi Farid H, et al. Family characteristics of individuals with risky sexual behaviors. Journal of Fundamentals of Mental Health. 2015;17(3):148–54.

[30] Kotchick BA, Shaffer A, Forehand R, Miller KS. Adolescent sexual risk behavior: a multi-system perspective. Clinical Psychology Review. 2001;21(4):493–519. doi: 10.1016/s0272-7358(99)00070-7 11413865

[31] Kaggwa MM, Arinaitwe I, Nduhuura E, Muwanguzi M, Kajjimu J, Kule M, et al. Prevalence and factors associated with depression and suicidal ideation during the COVID-19 pandemic among university students in Uganda: A cross-sectional study. Frontiers in Psychiatry. 2022;13:842466 doi: 10.3389/fpsyt.2022.842466 35492697 PMC9046690

[32] Kaggwa MM, Arinaitwe I, Muwanguzi M, Nduhuura E, Kajjimu J, Kule M, et al. Suicidal behaviours among Ugandan university students: A cross-sectional study. BMC Psychiatry. 2022;22(1):234. doi: 10.1186/s12888-022-03858-7 35365105 PMC8972906

[33] Najjuka SM, Checkwech G, Olum R, Ashaba S, Kaggwa MM. Depression, anxiety, and stress among Ugandan university students during the COVID-19 lockdown: An online survey. African Health Sciences. 2021;21(4):1533–43. doi: 10.4314/ahs.v21i4.6 35283951 PMC8889827

[34] Mamun MA, Al Mamun F, Hosen I, Hasan M, Rahman A, Jubayar AM, et al. Suicidality in Bangladeshi young adults during the COVID-19 pandemic: The role of behavioral factors, COVID-19 risk and fear, and mental health problems. Risk Management and Healthcare Policy. 2021;14:4051–61. doi: 10.2147/RMHP.S330282 34616192 PMC8488028

[35] Turchik JA, Walsh K, Marcus DK. Confirmatory validation of the factor structure and reliability of the Sexual Risk Survey in a large multiuniversity sample of U.S. students. International Journal of Sexual Health. 2015;27(2):93–105. doi: 10.1080/19317611.2014.944295

[36] World Health Organisation. Adverse Childhood Experiences International Questionnaire (ACE-IQ) 2020 [cited 2022]. https://www.who.int/publications/m/item/adverse-childhood-experiences-international-questionnaire-(ace-iq).

[37] Kidman R, Smith D, Piccolo LR, Kohler H-P. Psychometric evaluation of the Adverse Childhood Experience International Questionnaire (ACE-IQ) in Malawian adolescents. Child Abuse & Neglect. 2019;92:139–45. 10.1016/j.chiabu.2019.03.015. 30974257 PMC6513701

[38] Kidman R, Kohler H-P. Adverse childhood experiences, sexual debut and HIV testing among adolescents in a low-income high HIV-prevalence context. AIDS. 2019;33(14). 10.1097/QAD.0000000000002352 PMC6832840 31449094

[39] Kiburi SK, Molebatsi K, Obondo A, Kuria MW. Adverse childhood experiences among patients with substance use disorders at a referral psychiatric hospital in Kenya. BMC Psychiatry. 2018;18(1):197. doi: 10.1186/s12888-018-1780-1 29914409 PMC6007077

[40] Wang SC, Lui JHL, Vega G, Waldrop M, Garris J. The moderating effect of alcohol use on protective and risky sex behaviors among college students in the Southeast United States. Journal of American college health 2018;66(7):546–52. doi: 10.1080/07448481.2018.1431916 29405897

[41] Hahn AM, Simons RM, Simons JS. Childhood maltreatment and sexual risk taking: The mediating role of alexithymia. Archives of Sexual Behavior. 2016;45(1):53–62. doi: 10.1007/s10508-015-0591-4 26318149 PMC6555481

[42] Ntozi JP, Najjumba IM, Ahimbisibwe F, Ayiga N, Odwee J. Has the HIV/AIDS epidemic changed sexual behaviour of high risk groups in Uganda? African Health Sciences. 2003;3(3):107–16. 14676715 PMC2141609

[43] Gebresllasie F, Tsadik M, Berhane E. Potential predictors of risk sexual behavior among private college students in Mekelle City, North Ethiopia. The Pan African Medical Journal. 2017;28:151. doi: 10.11604/pamj.2017.28.151.5370 29564034 PMC5851670

[44] Tura G, Alemseged F, Dejene S. Risky sexual behavior and predisposing factors among students of Jimma University, Ethiopia. Ethiopian Journal of Health Sciences. 2012;22(3):170–80. 23209351 PMC3511895

[45] Shayo FK, Kalomo MH. Prevalence and correlates of sexual intercourse among sexually active in-school adolescents: An analysis of five sub-Sahara African countries for the adolescent’s sexual health policy implications. BMC Public Health. 2019;19(1):1285. doi: 10.1186/s12889-019-7632-1 31606038 PMC6790023

[46] McHaro RD, Olomi W, Mayaud P, Msuya SE. Risky sexual behaviours among young adults attending Higher Learning Institutions in Mbeya, Tanzania: implications for STIs and HIV preventive programs. AAS Open Research. 2021;3(41):41. 10.12688/aasopenres.13123.1 PMC10080207 37168604

[47] Okechi OS. The indigenous concept of sexuality in African tradition and globalization. Global Journal of Reproductive Medicine. 2018;6(1):1–5. 10.19080/GJORM.2018.06.555676.

[48] Leivo DN, Leonhard C, Johnson K, Carlson T. Relationship of high-risk sexual behaviors, sexual knowledge, and sexual satisfaction among African American college students: Toward a sex positive approach to STI prevention. Sexuality & Culture. 2021. doi: 10.1007/s12119-021-09884-z

[49] Menon JA, Mwaba SOC, Thankian K, Lwatula C. Risky sexual behaviour among university students. International STD Research & Reviews. 2016;4(1):1–7. doi: 10.9734/ISRR/2016/25462

[50] Somba MJ, Mbonile M, Obure J, Mahande MJ. Sexual behaviour, contraceptive knowledge and use among female undergraduates’ students of Muhimbili and Dar es Salaam Universities, Tanzania: a cross-sectional study. BMC Women’s Health. 2014;14(1):94. doi: 10.1186/1472-6874-14-94 25099502 PMC4126911

[51] Duby Z, Hartmann M, Montgomery ET, Colvin CJ, Mensch B, van der Straten A. Sexual scripting of heterosexual penile-anal intercourse amongst participants in an HIV prevention trial in South Africa, Uganda and Zimbabwe. Culture, Health & Sexuality. 2016;18(1):30–44. doi: 10.1080/13691058.2015.1064165 26223703 PMC4659730

[52] Duby Z, Hartmann M, Montgomery ET, Colvin CJ, Mensch B, van der Straten A. Perceptions and practice of heterosexual anal sex amongst VOICE participants in Uganda, Zimbabwe and South Africa. AIDS Research and Human Retroviruses. 2014;30(S1):A269–A. doi: 10.1089/aid.2014.5605.abstract

[53] Owen BN, Elmes J, Silhol R, Dang Q, McGowan I, Shacklett B, et al. How common and frequent is heterosexual anal intercourse among South Africans? A systematic review and meta-analysis. Journal of the International AIDS Society. 2017;19(1):21162. doi: 10.7448/IAS.20.1.21162 28364565 PMC5461120

[54] Keetile M. High-risk behaviors among adult men and women in Botswana: Implications for HIV/AIDS prevention efforts. SAHARA-J: Journal of Social Aspects of HIV/AIDS. 2014;11(1):158–66. doi: 10.1080/17290376.2014.960948 25293869 PMC4272173

[55] Ige OK, Ilesanmi OS, Adebayo AM. Sexual risk behaviours among young people with adverse childhood experiences in Ibadan, Nigeria. Greener Journal of Medical Sciences. 2012;2(3):70–6.

[56] Reach for Uganda. There is so much more than the nuclear family, even now 2020 [cited 2022 25/July/2022]. https://reachforuganda.org/there-is-so-much-more-than-the-nuclear-family-even-now/.

[57] Simeonova JI, Tzvetanova NV, Tzvetanova YI. Family factors and risk sexual behaviour in students aged 12–18. Journal of Biomedical and Clinical Research. 2015;7(1):31–6. doi: 10.1515/jbcr-2015-0122

[58] Lavielle-Sotomayor P, Jiménez-Valdez F, Vázquez-Rodríguez A, Aguirre-García M del C, Castillo-Trejo M, Vega-Mendoza S. [The impact of family characteristics in sexual risk behaviour of teens]. Revista medica del Instituto Mexicano del Seguro Social. 2014;52(1):38–43. 24625482

[59] Ali MM, Ajilore O. Risky sexual behavior and african-american youth: What is the role of family structure? Journal of Health Behavior & Public Health. 2011;1(1):30–40.

[60] Folkman S, Chesney MA, Pollack L, Phillips C. Stress, coping, and high-risk sexual behavior. Health Psychology. 1992;11(4):218–22. doi: 10.1037//0278-6133.11.4.218 1396489

[61] Song W, Qian X. Adverse childhood experiences and teen sexual behaviors: The role of self-regulation and school-related factors. Journal of School Health. 2020;90(11):830–41. doi: 10.1111/josh.12947 32929742

[62] Kaggwa MM, Nuwamanya S, Ashaba S, Rukundo GZ, Harms S. An adolescent’s use of veterinary medicines: A case report exploring addiction. Journal of Psychoactive Drugs. 2021;53(4):339–44. doi: 10.1080/02791072.2021.1873466 33432874

[63] Boothby N, Mugumya F, Ritterbusch AE, Wanican J, Bangirana CA, Pizatella AD, et al. Ugandan households: A study of parenting practices in three districts. Child Abuse and Neglect. 2017;67:157–73. doi: 10.1016/j.chiabu.2017.02.010 28273491

[64] Schuster MA, Bell RM, and Kanouse DE. The sexual practices of adolescent virgins: Genital sexual activities of high school students who have never had vaginal intercourse. American Journal of Public Health. 1996; 86(11):1570–1576. doi: 10.2105/ajph.86.11.1570 8916522 PMC1380691

[65] Yakubu I, Garmaroudi G, Sadeghi R, Tol A, Yekaninejad MS, Yidana A. Assessing the impact of an educational intervention program on sexual abstinence based on the health belief model amongst adolescent girls in Northern Ghana, a cluster randomised control trial. Reproductive Health. 2019;16(1):124. doi: 10.1186/s12978-019-0784-8 31416450 PMC6694566

[66] Champion VL, Skinner CS. The health belief model. Health Behavior and Health Education: Theory Research and Practice. 2008;4:45–65

[67] Bosnjak M, Ajzen I, Schmidt P. The theory of planned behavior: Selected recent advances and applications. Eur J Psychol. 2020;16(3):352–6. doi: 10.5964/ejop.v16i3.3107 33680187 PMC7909498

