# Supplementary material for: Risky sexual behaviours among Ugandan university students: A pilot study exploring the role of adverse childhood experiences, substance use history, and family environment
Source: PLoS One. 2022 Nov 16;17(11):e0277129. doi: 10.1371/journal.pone.0277129 (PMC9668123; doi:10.1371/journal.pone.0277129)
Supplement: S1 Table — (DOCX) [file pone.0277129.s001.docx]

### Sexual Risk Survey (SRS) Items and converting raw scores to ordinal categories

| **Question number** | **Items** | **Converting the raw scores into ordinal categorical scores** | | | | |
| --- | --- | --- | --- | --- | --- | --- |
|  |  | **0** | **1** | **2** | **3** | **4** |
| **F­_1_** | **Sexual risk taking with uncommitted partners** | | | | | |
| 8 | How many partners have you had sex with? | 0 | 1 | 2 | 3-6 | 7+ |
| 16 | How many people have you had sex with that you know but are not involved in any sort of relationship with (i.e., ‘‘friends with benefits’’, ‘‘fuck buddies’’)? | 0 | 1 | 2-3 | 4-5 | 6+ |
| 17 | How many times have you had sex with someone you don’t know well or just met? | 0 | 1 | 2-3 | 4 | 5+ |
| 19 | How many times have you had sex with a new partner before discussing sexual history, IV drug use, disease status and other current sexual partners? | 0 | 1-2 | 3-4 | 5-8 | 9+ |
| 20 | How many times (that you know of) have you had sex with someone who has had many sexual partners? | 0 | 1 | 2-3 | 4-9 | 10+ |
| 21 | How many partners (that you know of) have you had sex with who had been sexually active before you were with them but had not been tested for STIs/HIV? | 0 | 1 | 2-3 | 4 | 5+ |
| 22 | How many partners have you had sex with that you didn’t trust? | 0 | 1 | 2 | 3 | 4+ |
| 23 | How many times (that you know of) have you had sex with someone who was also engaging in sex with others during the same time period? | 0 | 1 | 2 | 3-4 | 5+ |
| **F_2_** | **Risky sex acts** | | | | | |
| 9 | How many times have you had vaginal intercourse without a latex or polyurethane condom? **Note:** Include times when you have used a lambskin or membrane condom? | 0 | 1-4 | 5-19 | 20-64 | 65+ |
| 10 | How many times have you had vaginal intercourse without protection against pregnancy? | 0 | 1-3 | 4-10 | 11-49 | 50+ |
| 11 | How many times have you given or received fellatio (oral sex on a man) without a condom? | 0 | 1-5 | 5-15 | 16-44 | 45+ |
| 12 | How many times have you given or received cunnilingus (oral sex on a woman) without a dental dam or ‘‘adequate protection’’? | 0 | 1-4 | 5-11 | 12-39 | 40+ |
| 18 | How many times have you or your partner used alcohol or drugs before or during sex? | 0 | 1-3 | 4-8 | 9-19 | 20+ |
| **F_3_** | **Impulsive sexual behaviors** | | | | | |
| 1 | How many partners have you engaged with in sexual behavior but NOT had sex? | 0 | 1 | 2–3 | 4 | 5+ |
| 2 | How many times have you left a social event with someone you just met? | 0 | 1 | 2–3 | 4 | 5+ |
| 3 | How many times have you ‘‘hooked up’’ (but NOT had sex) with someone you didn’t know or didn’t know well? | 0 | 1 | 2–3 | 4 | 5+ |
| 6 | How many times have you had an unexpected and unanticipated sexual experience? | 0 | 1 | 2 | 3-5 | 6+ |
| 7 | How many times have you had a sexual encounter you engaged in willingly but later regretted? | 0 | 1 | 2 | 3-4 | 5+ |
| **F_4_** | **Intent to engage in risky sexual behaviors** | | | | | |
| 4 | How many times have you gone out to bars/parties/social events with the intent of ‘‘hooking up’’ and engaging in sexual behavior but not having sex with someone? | 0 | 1-2 | 3-5 | 6-14 | 15+ |
| 5 | How many times have you gone out to bars/parties/ social events with the intent of ‘‘hooking up’’ and having sex with someone? | 0 | 1-2 | 3-5 | 6-19 | 20+ |
| **F_5_** | **Risky anal sex acts** | | | | | |
| 13 | How many times have you had anal sex without a condom? | 0 | 1-2 | 3-4 | 5-10 | 11+ |
| 14 | How many times have you or your partner engaged in anal penetration by a hand (‘‘fisting/fingering’’) or other object without a latex glove or condom followed by unprotected anal sex? | 0 | 1-2 | 3-5 | 6-10 | 11+ |
| 15 | How many times have you given or received analingus (oral stimulation of the anal region, ‘‘rimming’’) without a dental dam or ‘‘adequate protection’’? | 0 | 1-2 | 3-5 | 6-9 | 10+ |
